# Supplementary material for: Phage DisCo: targeted discovery of bacteriophages by co-culture
Source: mSystems. 2025 May 28;10(6):e01644-24. doi: 10.1128/msystems.01644-24 (PMC12172434; doi:10.1128/msystems.01644-24)
Supplement: Supplemental figures and tables — Figures S1 to S8; Tables S1 to S4. [file msystems.01644-24-s0001.docx]

**Supplemental Tables and Figures**

***Supplemental Table 1:*** Characterized phages and *E. coli* strains used to validate Phage DisCo

| **Characterized phages** | |
| --- | --- |
| **Phage** | **Receptor** |
| T4 | OmpC |
| U136B (12) | TolC |
| Bas37 (22) | Tsx |
| Bas10 (22) | PqqU |
| ***E. coli* BW25113 strains** (28) | |
| **Modification** | **Plasmid** |
| ∆*tolC* | pEB2-chlor-sGFP2 |
| ∆*tsx* | pEB2-chlor-mTurquoise2 |
| ∆*pqqU* | pEB2-chlor-mScarlet-I |


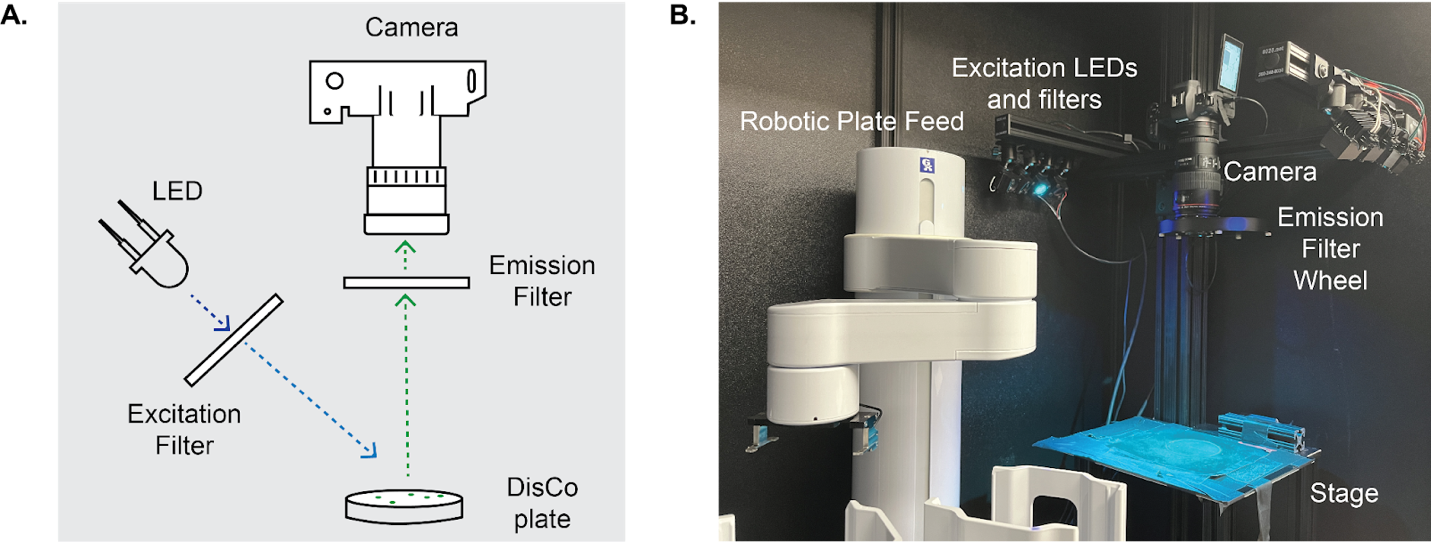


**Supplemental Figure 1: *Fluorescent imaging set up*. A.** Conceptual image of the bare bones needed for any macroscopic fluorescent imager. **B.** Image of custom set up with an array of excitation LEDs and filters, an emission filter wheel, and a robotic plate feed for high throughput imaging. Parts list for our imager in Supplemental Table 2.

***Supplemental Table 2:*** Parts list for custom fluorescence imager

| **Fluorescence** | | | |
| --- | --- | --- | --- |
| **Channel** | **Excitation LED** | **Excitation Filter** | **Emission Filter**  50mm diameter  Edmund optics  Format - X/Y  X - center wavelength (nm)  Y - width of band (nm) |
| Red | [Lime](https://www.luxeonstar.com/lime-567nm-sinkpad-ii-23mm-quad-led-module-1104lm) (567nm)  Luxeon Star SP-08-L1 | [562nm](https://www.edmundoptics.com.sg/p/562nm-cwl-25mm-dia-40nm-bandwidth-od-6-fluorescence-filter/21573/)  Edmond Optics #67-033 | [641/75](https://www.edmundoptics.com/p/641nm-cwl-50mm-dia-75nm-bandwidth-od-6-fluorescence-filter/21590/)  Edmund Optics #67-050 |
| Blue | [Royal blue](https://www.luxeonstar.com/royal-blue-448nm-sinkpad-ii-23mm-quad-led-module-3090mW) (448nm)  Luxeon Star SP-08-V4 | [438nm](https://www.edmundoptics.com/p/438nm-cwl-25mm-dia-24nm-bandwidth-od-6-fluorescence-filter/21566/)  Edmond Optics #67-026 | [483/31](https://www.edmundoptics.com/p/483nm-cwl-50mm-dia-31nm-bandwidth-od-6-fluorescence-filter/21582/)  Edmund Optics #67-042 |
| Green | [Cyan](https://www.luxeonstar.com/cyan-505nm-sinkpad-ii-23mm-quad-led-module-228lm) (505nm)  Luxeon Star SP-08-C2 | [494nm](https://www.edmundoptics.com/p/494nm-cwl-25mm-dia-20nm-bandwidth-od-6-fluorescence-filter/27204/)  Edmond Optics #84-96 | [540/50](https://www.edmundoptics.com/p/540nm-cwl-50mm-dia-50nm-bandwidth-od-6-fluorescence-filter/28802/)  Edmund Optics #86-366 |
| **Camera** | | | |
| [Canon EOS R](https://www.usa.canon.com/shop/p/eos-r?color=Black&type=New) (B&H Photo MFR #3075C002) | | | |
| **Lens** | | | |
| [Canon EF 100mm f2.8 Macro IS USM Lens](https://www.bhphotovideo.com/c/product/647011-USA/Canon_3554B002_EF_100mm_f_2_8L_Macro.html) (**B&H Photo** MFR #3554B002) | | | |


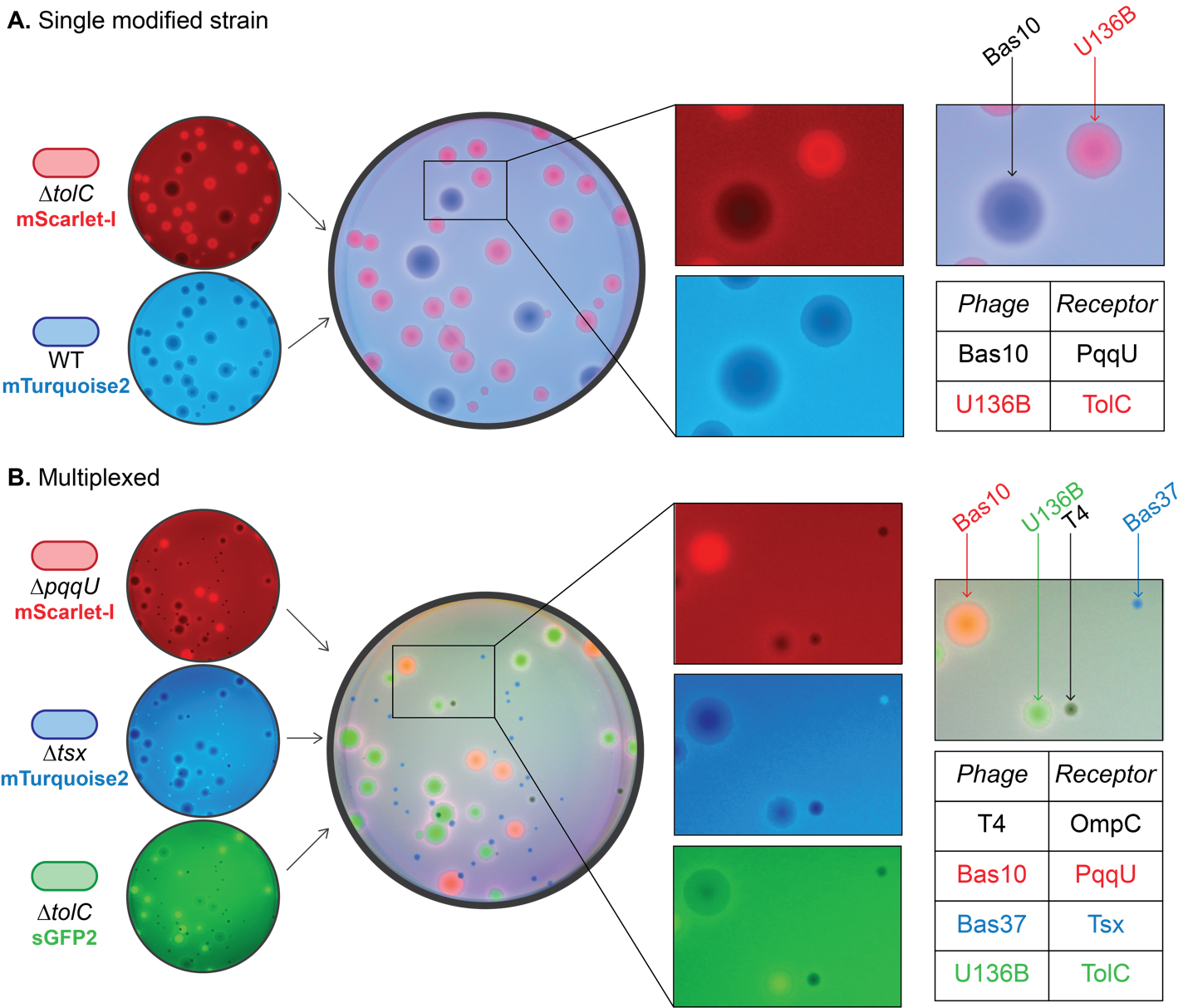


**Supplemental Figure 2: *Positive controls show expected plaque morphology on Phage DisCo plates*.** **A.** Two strain plate with a wildtype (WT) *E. coli* strain tagged blue and a *tolC* knockout strain (∆*tolC*) strain tagged red. As expected, Bas 10 plaques are dark (no fluorescent signal) while U136B plaques are red (red fluorescent signal) where the ∆*tolC* strain continues to grow. **B.** Multiplexed plate with three strains showing that, in this case, no WT strain is necessary and we can detect all three colored plaques. Phage T4, which does not require any of the individually knocked-out proteins for infection, lyses all three strains and leaves a dark plaque with no fluorescent signal. Phage U136B, which has been characterized to require the efflux protein TolC for infection (12), lyses all but the *tolC* knockout strain. Within the U136B plaques, the ∆*tolC* strain (tagged with GFP) alone is able to grow and therefore these plaques appear as green in the composite image. Similarly, phage Bas 37, which requires the Tsx protein, has a blue fluorescent signal corresponding to the ∆tsx strain, and phage Bas 10 which requires PqqU and has a red fluorescent signal corresponding to ∆*pqqU* (22).


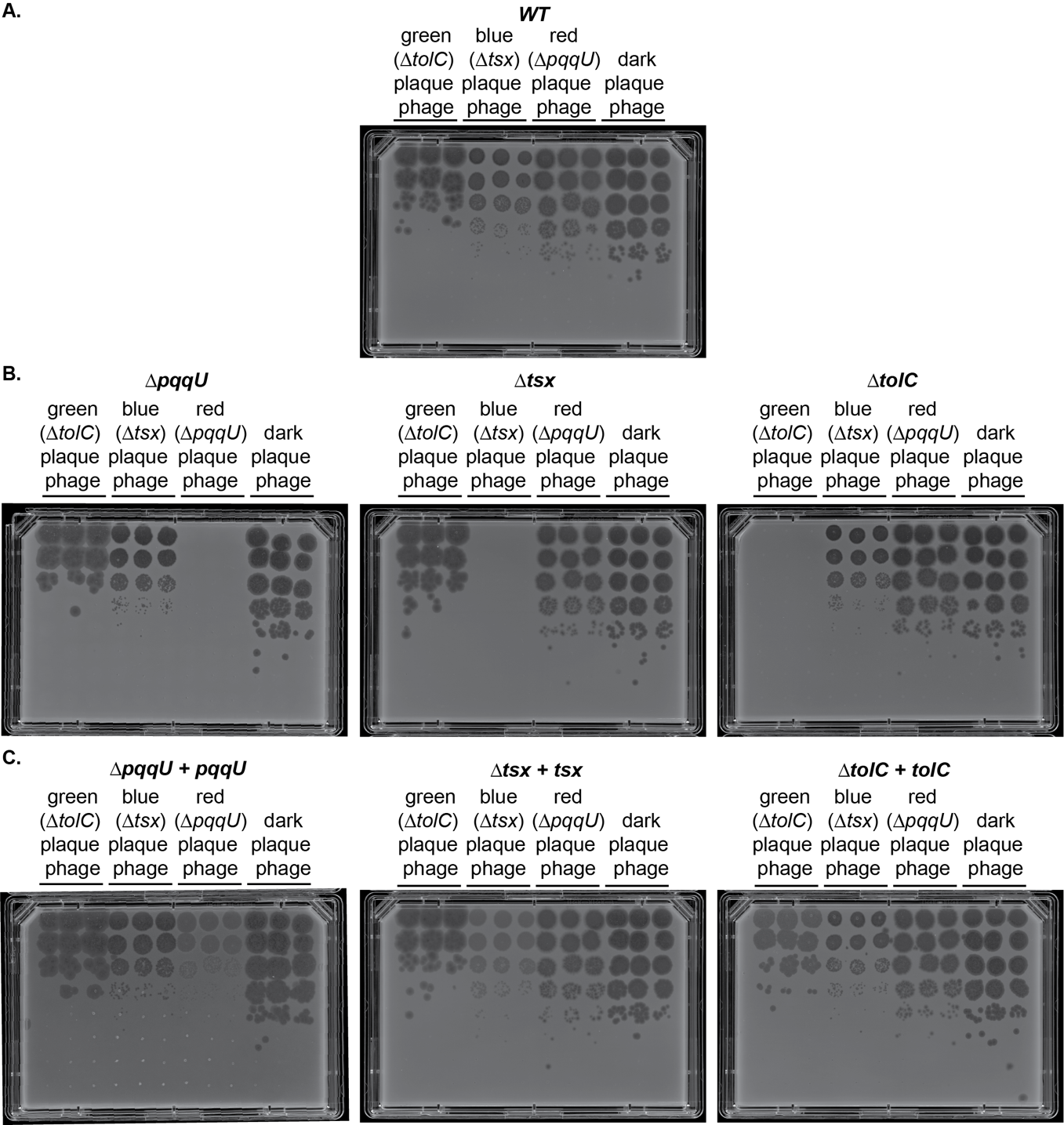


**Supplemental Figure 3: *Plaque assays of each phage picked from Figure 2A plated on all seven potential hosts in monoculture which were used to create graphs in Figure 2B*.** **A.** All phages plated on the wildtype (WT) lawn. **B.** All phages plated on each of the knockout strains (*∆pqqU*, *∆tsx*, and *∆tolC*) in monoculture. **C.** All phages plated on each of the complemented strains (*∆pqqU + pqqU*, *∆tsx + tsx*, and *∆tolC + tolC*) in monoculture. For all plates, the bacterial strain on the lawn is listed above the image in bold font. Three separate serial dilutions of each phage were made to provide biological replicates. 2uL of each dilution was spotted on each plate using a Gilson Platemaster (96 well pipette). The dilutions shown represent the -3 through the -10 dilutions.


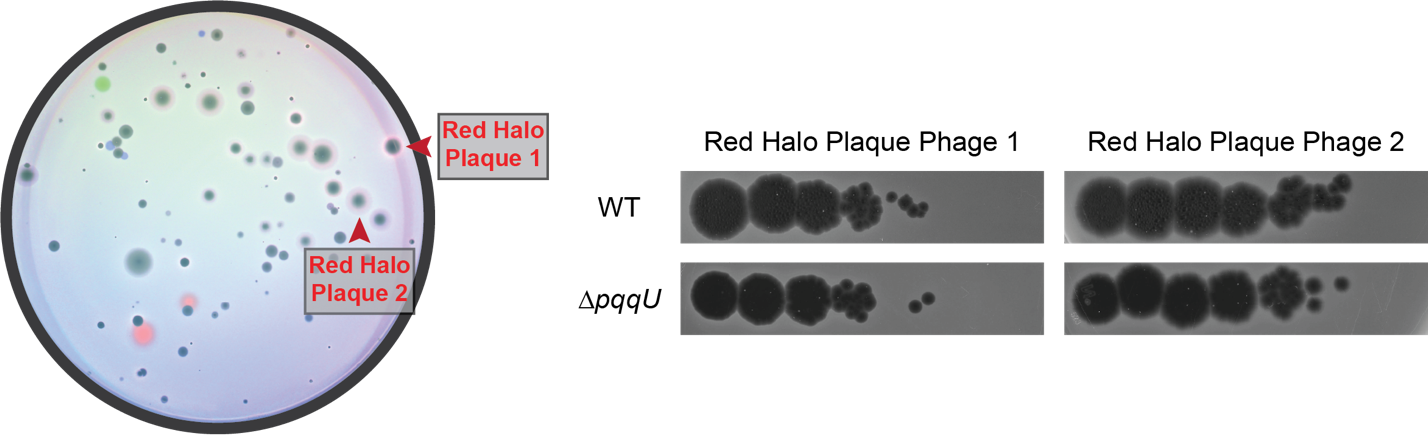


**Supplemental Figure 4:** *In this case, plaques with red halos do not have receptor specific properties.* The screening plate on the left was made using the three strains listed in Supp. Table 1 plus a sample of filtered wastewater. Plaques with red rings, or halos, from the screening plate were picked and plated on monoculture lawns of either the wildtype (WT) *E. coli* BW25113 strain or the *pqqU* knockout.

***Supplemental Table 3:*** Phages and *E. coli* strains used to validate phage defense Phage DisCo

| **GmrSD Experiments** | |
| --- | --- |
| **Characterized phages** | |
| **Phage** | **GmrSD susceptibility** |
| T4 | No |
| T4 ∆IPI (25) | Yes |
| ***E. coli* MG1655 strains** | |
| **Defense system** | **Plasmid** |
| - | pBR322-mWatermelon |
| pSC-gmrSD | pBR322-mScarlet-I |
| **CBASS Experiments** | |
| ***E. coli* MG1655 strains** | |
| - | pEB2-chlor-mTurquoise2 |
| pBADS-CBASS_EcCdnD_4TM ([Addgene #224395](https://www.addgene.org/224395/)) | pEB2-chlor-mScarlet-I |
| **BstA Experiments** | |
| ***Salmonella enterica* LT2 strains** (26) | |
| BstA-STOP | pEB2-chlor-mTurquoise2 |
| BstA | pEB2-chlor-mScarlet-I |
| **Characterized Phages** | |
| Phage | BstA susceptibility |
| BTP1 | No |
| BTP1∆*bstA* (26) | Yes |


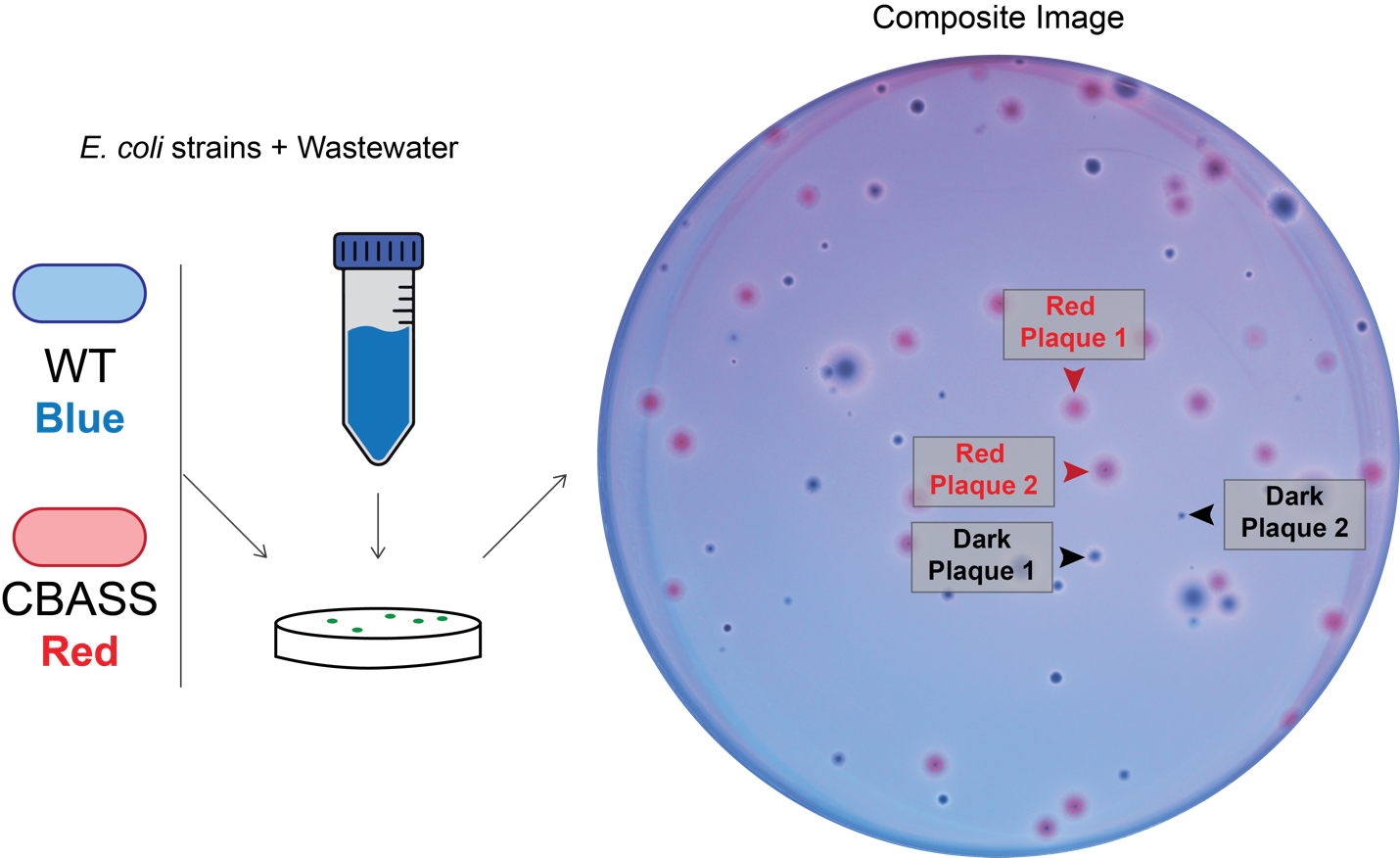


**Supplemental Figure 5: *CBASS screening plate shows many hits in wastewater sample*.** Here red plaques are predicted to be interacting with the CBASS defense system and dark plaques are not. The highlighted plaques were picked, replicated, and plated on monoculture lawns to make the images shown in Figure 3E.


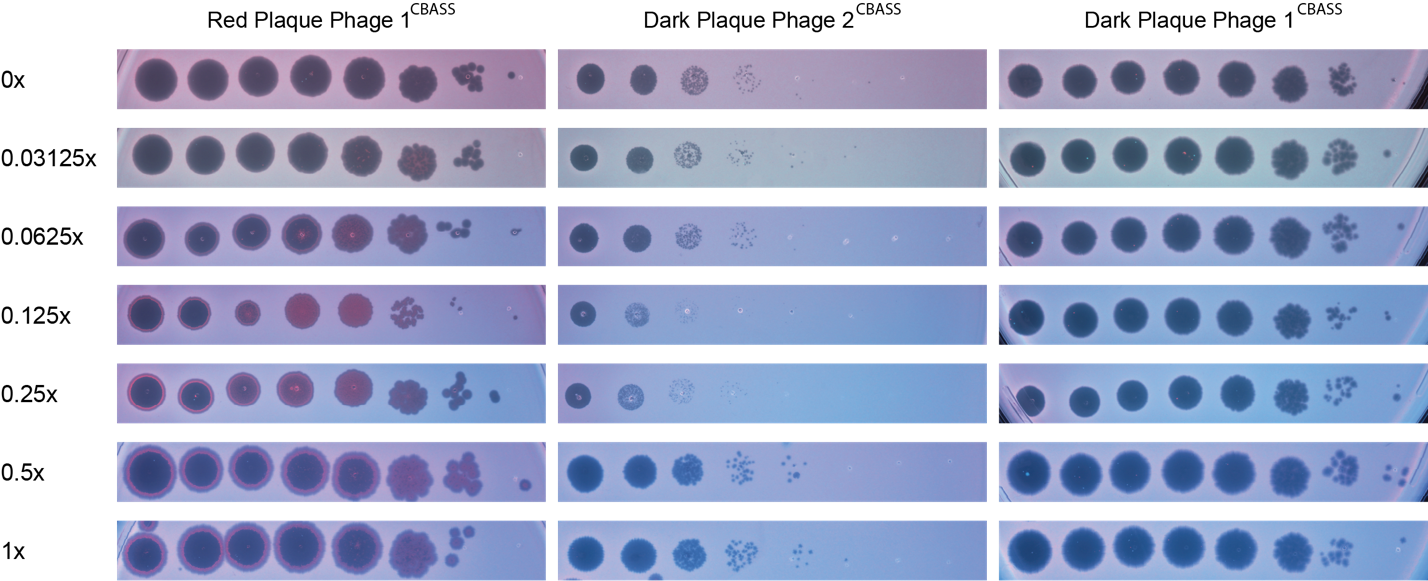


**Supplemental Figure 6: *2x serial dilutions show that no concentration of inducer tested leads to a red halo around the plaques made by Dark Plaque Phage 1^CBASS^ as expected for a CBASS sensitive phage.*** The 1x concentration is 0.2% arabinose and was the concentration used for all other experiments. Red Plaque Phage 1^CBASS^ and Dark Plaque Phage 2^CBASS^ were used as controls for plaques expected to have a red halo and not have a red halo respectively.


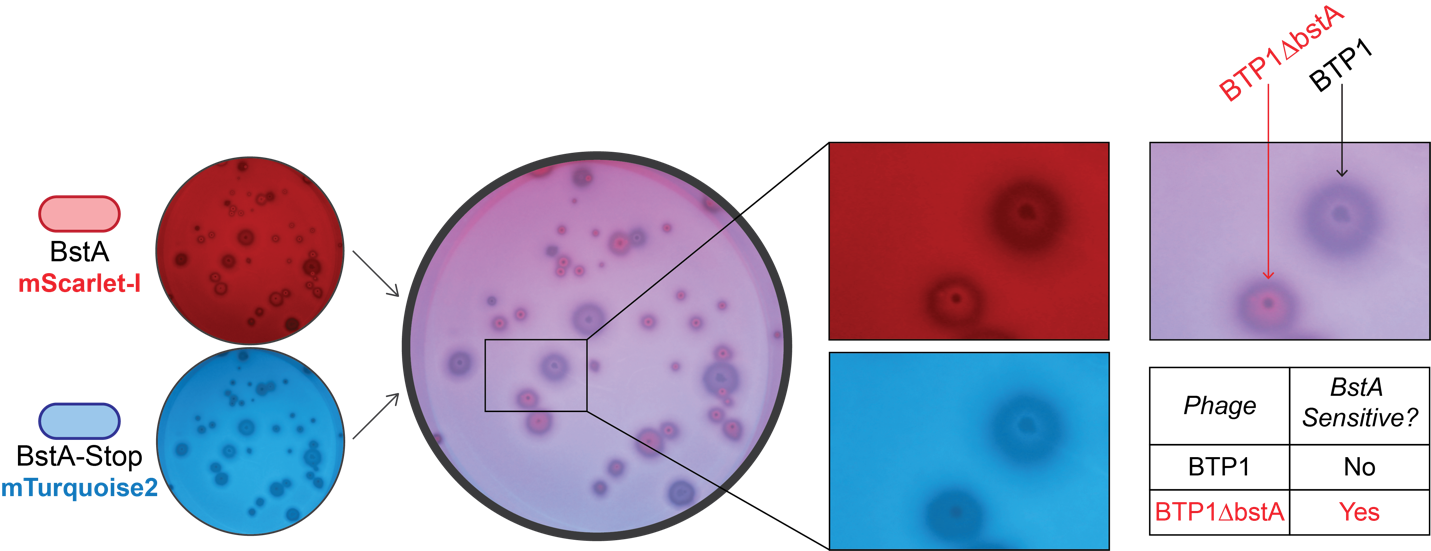


**Supplemental Figure 7: *BstA control plate shows that lysogenic phages are also detectable using Phage DisCo*.** Shown here is a control plate with two *Salmonella enterica* LT2 strains. BstA is a prophage encoded phage defense system. BTP1 is expected to be able to lyse a cell with BstA on the chromosome while BTP1∆*bstA* is not (26). The blue strain contains a BstA sequence with an early stop codon while the red contains a functioning BstA defense system. As is anticipated, BTP1makes a dark plaque while BTP1∆*bstA* has red fluorescent signal within the plaque.

***Supplemental Table 4:*** Summary of plasmids used in Phage DisCo experiments and fluorescent detection conditions for each one

| Plasmid | Description | Selectable Marker | Filters Used for Detection (EX-EM) |
| --- | --- | --- | --- |
| pEB2-chlor-sGFP2 | Derivative of Addgene #104007 where Switch KanR for CmR and mScarlet-I for sGFP2 (38) | CmR | 494nm – 540/50nm |
| pEB2-chlor-mTurquoise2 | Derivative of Addgene #104007 where Switch KanR for CmR and mScarlet-I for mTurquoise2 (38) | CmR | 438nm – 483/31nm |
| pEB2-chlor-mScarlet-I | Derivative of Addgene #104007 where Switch KanR for CmR | CmR | 562nm – 641/75nm |
| pBR322-mWatermelon | ORI and rop from NEB #N3033S with KanR and mWatermelon (49) | KanR | 494nm – 540/50nm |
| pBR322-mScarlet-I | ORI and rop from NEB #N3033S with KanR and mScarlet-I (38) | KanR | 562nm – 641/75nm |


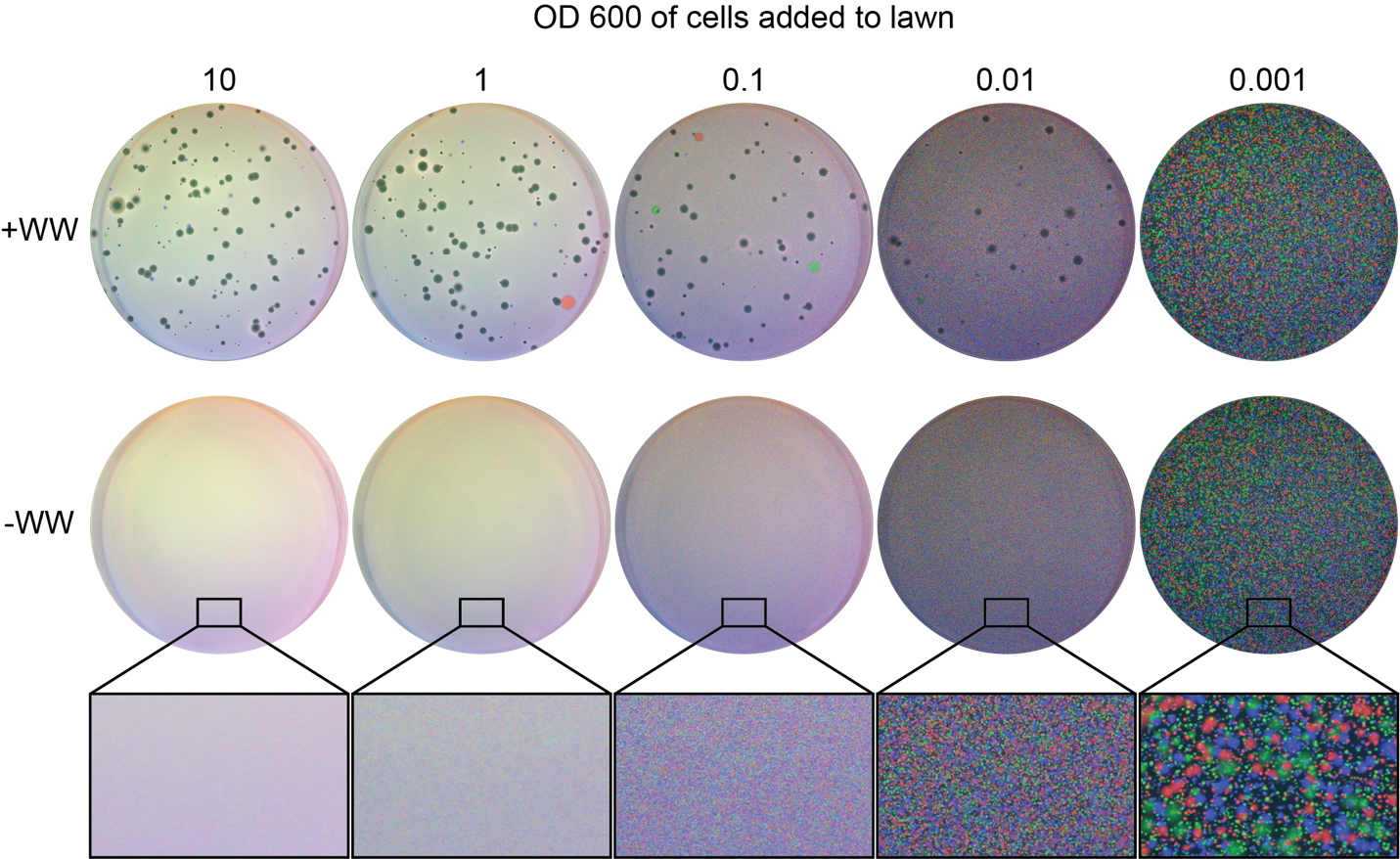


**Supplemental Figure 8: *Increasing starting cell inoculum leads to more homogenous top agar lawns.*** All plates were made with *E. coli* strains listed in Supp. Table 1, and the top row has 300uL of wastewater (WW) added as well. Although cells at OD=10 yield the smoothest lawn, fluorescent plaques are visible with 100x fewer cells on the plate.

49. Gohil K, Wu S-Y, Takahashi-Yamashiro K, Shen Y, Campbell RE. 2023. Biosensor optimization using a Förster resonance energy transfer pair based on mScarlet red fluorescent protein and an mScarlet-derived green fluorescent protein. ACS Sens 8:587–597. https://doi.org/10.1021/acssensors.2c01730
